# Exposures to combustion sources near military operations in Iraq and Afghanistan using satellite observations

**DOI:** 10.1038/s41370-025-00804-z

**Published:** 2025-10-10

**Authors:** Meredith Franklin, Xiaozhe Yin, Anna Korpak, Paul D. Blanc, Petros Koutrakis, Eric Garshick

**Affiliations:** 1 Department of Statistical Sciences and School of the Environment, University of Toronto, Toronto, ON Canada; 2 Department of Environmental Health, Harvard T.H. Chan School of Public Health, Boston, MA USA; 3 U.S. Department of Veterans Affairs, Seattle Epidemiologic Research and Information Center, VA Puget Sound Health Care System, Seattle, WA USA; 4 U.S. Department of Veterans Affairs, VA Healthcare System San Francisco, San Francisco, CA USA; 5 Department of Medicine, Division of Occupational, Environmental and Climate Medicine, University of California San Francisco, San Francisco, CA USA; 6 U.S. Department of Veterans Affairs, VA Healthcare System Boston, Boston, MA USA; 7 Harvard Medical School, Boston, MA USA

**Keywords:** Remote sensing, Combustion-related exposures, Occupational exposures, Military deployment, Veteran health

## Abstract

**Background:**

U.S. military personnel deployed to Afghanistan and Iraq were stationed on bases impacted by airborne hazards including emissions from combustion sources. Due to limited environmental monitoring during military operations, exposure levels remain poorly characterized.

**Objective:**

We used satellite observations to identify the locations and persistence of combustion sources on and near military bases in Afghanistan and Iraq from 2002 to 2012, the peak period of open-air combustion.

**Methods:**

Daily fire detections from the Moderate Resolution Imaging Spectroradiometer (MODIS) were clustered using density-based methods to identify persistent burning within 5 km of bases. Validation was conducted using military imagery and Google Earth. A sensitivity analysis compared MODIS fire detections to those from the newer Visible Infrared Imaging Spectroradiometer (VIIRS) at a civilian burn pit in Djibouti.

**Results:**

MODIS detected 285,810 fires in Iraq and 3702 in Afghanistan. Clustering identified 398 bases in Iraq and 122 in Afghanistan with burning nearby. In Iraq, persistent clusters were linked to oil and gas flares, while smaller clusters on bases in both countries were consistent with burn pits. MODIS and VIIRS both detected the Djibouti burn pit, but VIIRS recorded three times more fire detections, highlighting its sensitivity in detecting biomass and waste burning.

**Impact:**

- This study is the first to use satellite fire detections to objectively map and quantify combustion sources at U.S. military bases in Iraq and Afghanistan during 2002–2012. By applying density-based clustering to MODIS data and validating with high-resolution imagery, we identified persistent burning patterns near and on bases. This approach overcomes the limitations of self-reported exposure data and provides a reproducible framework for assessing deployment-related combustion exposures. The findings highlight both the utility and limitations of MODIS and demonstrate the potential of satellite observations for Veteran health research.

## Introduction

During land-based deployment in Iraq and Afghanistan, U.S. military personnel were potentially exposed to airborne pollutants from a variety of combustion sources [1, 2]. A prominent contributor on or near bases was the use of open-air burn pits to dispose of mixed waste materials [3–5]. This practice began with the onset of Operation Enduring Freedom in 2001 (Afghanistan), Operation Iraqi Freedom in 2003 (Iraq), and continued through at least 2011–2012 as the U.S. military began withdrawing from Iraq. Although incinerators were gradually introduced during this period, they typically supplemented rather than fully replaced open-air burning. Reports suggest that some burn pits remained in use after 2012, but on a much smaller scale [6].

In addition to on-base sources, southeastern Iraq, where many bases were concentrated, has long experienced significant air pollution from gas flaring associated with oil and gas operations [7].

Recognizing the potential health risks from these exposures, the U.S. Congress passed the Veterans Burn Pits Exposure Recognition Act in 2021, followed by the Promise to Address Comprehensive Toxics (PACT) Act in 2022. These laws expanded healthcare benefits for Veterans exposed to airborne hazards during deployment. However, efforts to evaluate long-term health effects have been hindered by the lack of systematic records documenting combustion activities in and around military installations, making exposure assessments particularly challenging.

Both on-base and off-base combustion-related emissions contributed significantly to deployment-era air pollution exposures and have been linked to adverse health outcomes [1, 3, 8, 9]. On-base combustion sources included not only  burn pits but also diesel-powered generators and vehicle exhaust. At Joint Base Balad, Iraq, air monitoring in 2007 identified burn pits as a major contributor to ambient air pollution [3]. A source apportionment study of these data estimated  that burn pit emissions accounted for approximately 75% of total exposure to toxic polychlorinated dibenzo-p-dioxin (PCDD) and polychlorinated dibenzofuran (PCDF) [10]. Experimental simulations further demonstrated that open-air burn pits emitted PCDD/PCDF at concentrations nearly 50 times higher than covered burn boxes, due to incomplete combustion and persistent smoldering [11]. Supporting these findings, air samples taken near burn pits and incinerators at Bagram Airfield, Afghanistan between 2005-2012 found the highest particulate matter (PM) concentrations near the solid waste disposal facility [12, 13].

Off-base sources also played an important role in shaping exposures. These included regional oil and gas flaring, industrial emissions, and local waste burning. Flaring from oil and gas operations, an understudied military exposure, releases PM and other harmful air pollutants [14]. Iraq is the world’s second -highest contributor to global flaring volume, burning nearly 18 billion cubic meters of gas annually [7].

The combined impact of both on-base and off-base combustion sources underscores the complexity of airborne exposures during military deployment. In the absence of systematic ground-level environmental monitoring in Iraq and Afghanistan, Earth-observing satellites offer a novel alternative for reconstructing historical burning activity. Remote sensing data, unlike traditional military or environmental records, allow for consistent, retrospective analysis of combustion sources over broad geographic areas and time periods. Two satellite instruments commonly used for fire detection are the Moderate Resolution Imaging Spectroradiometer (MODIS) [15] and the Visible Infrared Imaging Spectroradiometer (VIIRS) [16].

MODIS, with its 1 km spatial resolution, was the only available fire detection dataset available during the peak military deployment period in Afghanistan and Iraq (2002–2012). The launch of VIIRS in 2012 improved fire detection capabilities by providing higher spatial resolution (375 m) and greater sensitivity to smaller and lower temperature fires [17]. As a result, satellite-based fire detection now offers a powerful tool for reconstructing historical combustion activity and estimating deployment-related environmental exposures.

In this study, we hypothesized that persistent combustion sources in Iraq and Afghanistan during post-9/11 military operations (2002–2012) could be systematically identified using daily MODIS fire detections. Specifically, we tested whether spatial clustering of satellite fire detections could reveal patterns of persistent fire activity on and near military bases, thereby supporting assessments of potential exposure risks among deployed military personnel. To evaluate this, we applied a previously developed spatial clustering method [18] to quantify the geographic persistence of combustion relative to known base locations. We validated satellite-detected fire locations by examining fire clusters within 5 km of military bases using high-resolution georeferenced military imagery and Google Earth. To further assess the specificity of MODIS detections, we conducted a sensitivity analysis comparing MODIS and VIIRS active fire detections at a known civilian burn pit near the US military base Camp Lemonnier, Djibouti. This allowed us to evaluate the strengths and limitations of MODIS for detecting various types of combustion in military contexts and to develop metrics of fire exposure relevant to Veteran health.

## Materials and methods

### Satellite data

The National Aeronautics and Space Administration (NASA) Moderate Resolution Imaging Spectroradiometer (MODIS) instrument is on two satellites, Terra (local overpass between 10:00 and 13:00) and Aqua (local overpass between 12:00 and 15:00). MODIS Collection 6.1 (C6.1) active fire products, MOD14 (Terra) and MYD14 (Aqua), were acquired from NASA’s Fire Information for Resource Management System (FIRMS). The MODIS active fire algorithm detects thermal anomalies at 3.9 µm and 11 µm wavelengths, identifying heat sources such as biomass burning, volcanoes and gas flares (MODIS User Guide, 2021). The algorithm identifies the central longitude and latitude of a 1 × 1 km pixel containing one or more actively burning fires at the time of satellite overpass [19]. In addition to fire locations, the MODIS active fire product provides fire radiative power (FRP), which quantifies the rate at which a fire emits thermal radiation, expressed in megawatts (MW) [20]. FRP is widely used as a proxy for fire intensity, intensity and provides critical information about combustion dynamics. Specifically, FRP is directly proportional to the rate of biomass consumption, making it a valuable indicator for estimating emissions of smoke, particulate matter, and trace gases [21]. FRP reflects both the temperature and the size of the actively burning area and is calculated as the product of the fourth power of temperature, burning area, and the Stefan-Boltzmann constant. This means that a small, very hot fire can produce the same FRP as a larger, cooler one, making FRP a composite metric of fire behavior.

MODIS C6.1 can detect active fires covering as little as 0.01–0.1% of a 1 × 1 km pixel when fire temperatures exceed 1000 K [19] and is capable of detecting FRP as low as 2.5 MW at nadir [17]. However, detection sensitivity decreases for smaller or lower-temperature fires.

Wildfires and other vegetation burns typically produce temperatures ranging from 600 to 1200 K, while open-air waste burning generally occurs at lower temperatures (500 K to below 1000 K), especially under smoldering conditions rather than flaming combustion [11]. In contrast, industrial waste burning and oil and gas flaring occur at much higher temperatures, often exceeding 1600 K [18]. The distinction between flaming and smoldering combustion is critical as they produce significantly different air pollutant emissions, both in chemical composition and toxicity. Furthermore, the nature and temperature of combustion are strongly influenced by the composition of materials burned. Until around 2009, U.S. military burn pits frequently included not only food waste and general refuse but also hazardous and high-energy-content materials such as petroleum products, tires, plastics, batteries, medical waste, aerosol cans, paints, solvents, pesticides, and electronic equipment, as well as munitions and compressed gas cylinders [3, 4, 6]. Plastics and petroleum-based materials can produce high-temperature flames during brief flaming phases, but their incomplete combustion in open pits often leads to extended smoldering, which emits lower radiant energy and therefore lower fire radiative power (FRP). In contrast, materials like tires or oil-based waste may generate intense but localized heat, while others like medical waste or munitions may create short bursts of high temperature but do not contribute to sustained high FRP due to rapid consumption or fragmentation [10, 11].

The MODIS active fire algorithm assigns each detection a confidence value ranging from 0-100 based on factors influencing retrieval. These include the strength of the thermal signal (e.g., temperature), the spatial variability of the thermal source relative to surrounding pixels, surface brightness, cloud cover, and the sensor’s viewing angle. The confidence values are classified as low (0–30%), nominal (30–80%), and high (80–100%) [19]. We used detected fire observations with all confidence levels because we did not want to eliminate smoldering fires, which are often classified with low confidence due to their lower burning temperatures [22].

Due to the MODIS scan geometry and the curvature of the Earth, the size and shape of MODIS pixels become distorted from the center to the edge of the swath. This phenomenon, known as the “bow tie” effect, causes pixels to stretch in the longitudinal direction, leading to overlapping areas between consecutive scans near the swath edges [23]. If our study area includes some scan overlaps, it could result in a modest overestimation of the number of fires detected. To mitigate this potential bias, we removed detections identified as noise using a hierarchical density-based clustering analysis (HDBSCAN), described below.

In a joint venture between NASA and the National Oceanic and Atmospheric Administration (NOAA), the Visible Infrared Imaging Radiometer Suite (VIIRS) onboard the Suomi National Polar-orbiting Partnership (S-NPP) satellite began collecting fire observations at 375 m resolution in early 2012. The VIIRS 375 m active fire data, also acquired from FIRMS, can theoretically detect many more small and cool fires than the 1 km MODIS active fire data, as shown in a study of agriculture burning in Africa [17]. Furthermore, VIIRS has reduced bow tie effect compared to MODIS.

This study focuses on MODIS-observed active fire detections in Iraq and Afghanistan from 2002 to 2012, the period when burn pits were most commonly in operation on and near military bases. We conducted a sensitivity analysis using VIIRS active fire observations to assess the robustness of our findings.

### Statistical methods

Satellite active fire detections are sub-pixel in nature, meaning they represent fire-affected areas smaller than the native resolution of the satellite’s sensor and are georeferenced as points within the nominal resolution of the detection algorithm. As such, spatial point clustering methods facilitate the identification of persistent burning locations by grouping nearby points together. Our previous work identifying oil and gas flaring sources from VIIRS [18] used HDBSCAN because it has the advantage of allowing for irregularly shaped clusters and classifies aberrant fire detections as “noise” if they do not meet the threshold of being within a cluster. We applied the same approach here, using HDBSCAN to analyze MODIS fire detections, identify clusters, and filter out aberrant “noise” fires. HDBSCAN also provides a membership probability for each observation, which quantifies the strength of association between a point (fire observation) and the cluster to which it has been assigned. Values range from 0 to 1, with higher values indicating that the detected fire lies close to the dense core of the cluster and is strongly representative of that cluster. Lower values suggest that the point is on the edge of the cluster or less characteristic of the dense region and may be more weakly associated. In this study, we used the membership probability to assess the cohesiveness and confidence of each clustered fire detection, providing additional information about the robustness of the identified combustion patterns near military bases.

To account for the potential of bases moving and to characterize the temporal persistence of combustion activity, we applied HDBSCAN separately for each year, tuning the algorithm to have a minimum number of points per cluster (minPts), denoted as k. The value of k ranged from 3 to 8 for Iraq and 2 to 8 for Afghanistan and Djibouti. To determine the optimal k for each year, we systematically evaluated different values and selected the one that minimized the number of noise points while maintaining meaningful cluster structures. Through this process we avoided excessive fragmentation (k too small) and overly large clusters (k too large).

### Geospatially linking MODIS fire clusters to military bases

**Fig. 1:**
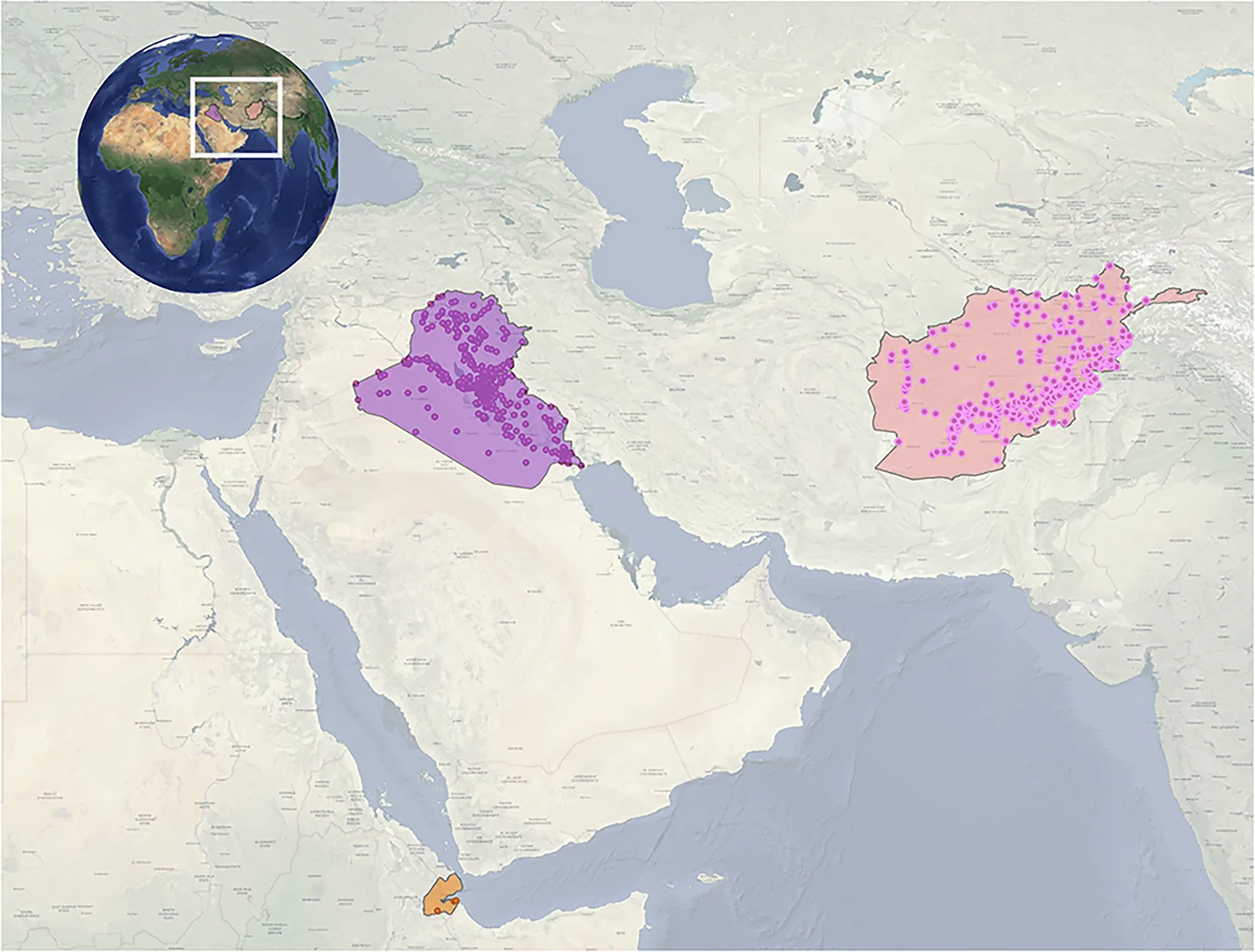
Map of the study region showing U.S. military base locations. The outlines of Afghanistan (pink), Iraq (purple), and Djibouti (orange) are shown, with military bases indicated by dots in corresponding colors.

Geocoded military base locations were sourced from ongoing research under Veteran Affairs (VA) Cooperative Studies Program (CSP) #595, “Pulmonary Health and Deployment to Southwest Asia and Afghanistan” (NCT02825654), also known as SHADE (Service and Health among Deployed Veterans). The lists of geocoded bases were compiled by the US Department of Defense, the National Geospatial Intelligence Agency, and the US Airforce 14^th^ Weather Squadron (Fig. 1). To associate the clustered fires with military bases, we created 5 km spatial buffers around each site to account for potential geolocation errors in both the satellite retrievals and base coordinates. Since MODIS fire detections are reported as point locations, their true position may be displaced within a 1 km grid cell due to sensor viewing angles, terrain effects, and retrieval uncertainty. Similarly, the precision of the base geocodes varied by source, with some coordinates representing the center of a large facilities rather than specific fire activity areas.

The use of 5 km buffers was further justified by the fact our dataset included a few large bases and complete perimeter data was not available, making it difficult to precisely determine fire locations within base boundaries. Additionally, many bases were in close proximity to one another. To refine the analysis we calculated the distances between bases, identifying those that were within 5 km of each other and reporting their associated fire clusters accordingly.

With the annual clusters, we were able to track year-by-year persistence in clustered fire activity within 5 km of each base, enabling the construction of annual timelines of combustion. Summary statistics were compiled to describe the number of fires, the number of clusters and the number of years with observed clusters for each base.

### Validation and sensitivity analysis

To validate the locations of bases as well as the clustered MODIS fires identified within 5 km of their geolocations, we obtained georeferenced imagery of major bases in Iraq and Afghanistan from the Buckeye system. The Buckeye system is a high-resolution aerial imaging system developed by the U.S. Army Geospatial Center to provide detailed geospatial information for military operations. The images were processed and geospatially integrated to GeoTIFFs. We used QGIS 3.14 on a secure VA server to thoroughly check each base by superimposing the base and clustered fire detections on each GeoTIFF. While we cannot publish these images, we summarize what we found, and where possible used GoogleEarth to present our findings.

Finally, to assess the sensitivity of MODIS in detecting low-temperature, smoldering fires, an area where it has shown limitations, we conducted an analysis focused on a known active civilian burn pit near Camp Lemonnier, Djibouti. We examined data from 2016 to 2023, a period during which the burn pit was active and both MODIS and the more sensitive, higher -resolution VIIRS datasets were available for comparison.

All data analyses and modeling were conducted using R 4.4.2.

## Results

### MODIS active fire detections

**Fig. 2:**
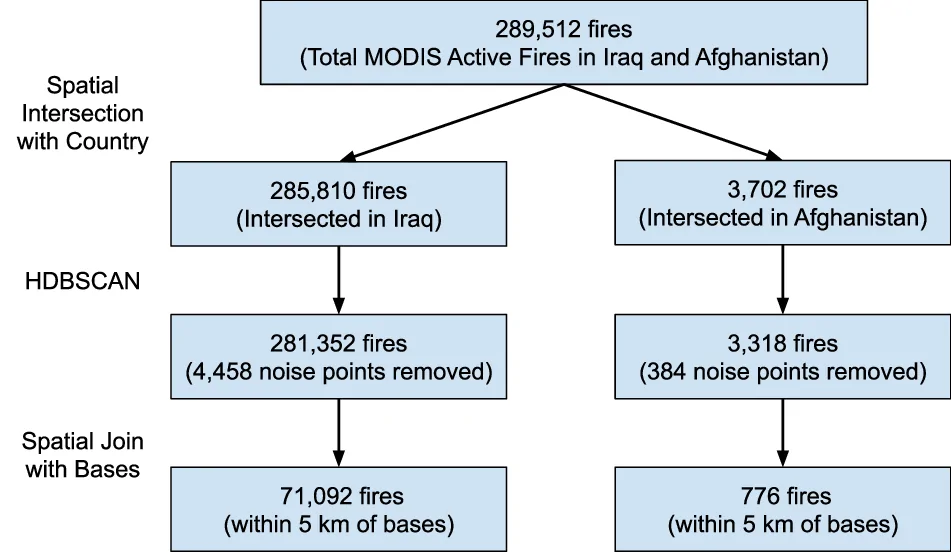
Flow diagram of MODIS Active Fire data processing steps to identify persistent burning sources within 5 km of military base locations in Iraq and Afghanistan between 2002 and 2012.

There were 285,810 MODIS active fire detections in Iraq and 3702 in Afghanistan from 2002 through 2012 (Fig. 2). In both countries, the distributions of retrieval confidence were skewed, with most values concentrated on the right towards higher confidence. The median retrieval confidence was 77 in Iraq (IQR = 26) and 69 in Afghanistan (IQR = 30). The mode (i.e., the most frequent) retrieval confidence in both countries was 100. The distributions of FRP were also skewed, but with the data concentrated at lower values. The median FRP was 21.6 MW (IQR = 24.8 MW) in Iraq and 19.9 MW (IQR = 30.4 MW) in Afghanistan. The higher median FRP in Iraq is likely due to the dominance of hotter industrial combustion sources like oil and gas flaring. The higher FRP IQR in Afghanistan suggests a greater mix of fire sources and heterogeneous topography, which may reduce detection particularly of lower-temperature fires.

### HDBSCAN results

After stratifying all MODIS fires by year and applying HDBSCAN, we distinguished persistent burn locations (fire observations that were part of clusters) from aberrant burning occurrences, which were classified as noise points (Fig. 2).

In Iraq, the tuned value of k (minimum number of fires in a cluster) was 5 in all years except for 2003 when it was 6. With this specification, noise observations accounted for 0.9% to 2.5% (overall 1.6%) of the total number of detections, leaving 281,352 fires that were clustered (Fig. 1). The number of annual clusters ranged from 92 to 192, and the number of fires per cluster was highly skewed, with medians ranging from 12 to 27.

We observed that the number of fires remained relatively stable from 2004 through 2009, with annual totals ranging from approximately 23,000 to 26,000 (Table 1). This consistency likely reflects the sustained use of burn pits and other combustion sources throughout the main years of U.S. operations. Notably, there was a peak in the number of clusters in 2010, followed by increases in total fire counts in both 2011 and 2012, reaching 32,256 and 36,776, respectively. These later peaks may correspond to heightened activity related to base closures and waste disposal as the U.S. military began its formal withdrawal from Iraq. In 2012, the year after withdrawal from Iraq many fires were still detected. It is possible that intensified clean-up efforts, increased burning of remaining waste, or reactivation of burn pits contributed to these elevated fire counts. In addition, Iraq’s substantial oil and gas infrastructure, particularly in the south, showed significant increases in flaring after 2010 (IEA Iraq 2000-2030 flaring trends) [24].

**Table 1:** Annual Summary Statistics of MODIS Active Fire Observations, Clusters, Noise, and Median Number of Fires per Cluster for Iraq and Afghanistan.

Iraq				Afghanistan		
Year	Clustered Fires *N* (Noise %)	Clusters *N*	Fires^a^/cluster N (IQR)	Clustered Fires N (Noise %)	Clusters *N*	Fires/cluster *N* (IQR)
2002	15,856 (2.5%)	117	16 (64)	196 (10.5%)	38	4 (3)
2003	18,186 (2.5%)	125	18 (71)	396 (15.6%)	70	4 (4)
2004	26,534 (1.0%)	114	16 (217)	261 (13.6%)	45	5 (4)
2005	23,417 (1.2%)	92	27 (260)	173 (10.8%)	24	5 (4)
2006	25,021 (0.9%)	106	12 (160)	340 (15.6%)	53	5 (3)
2007	25,629 (1.2%)	147	12 (33)	222 (17.8%)	38	5 (4)
2008	25,932 (1.5%)	153	14 (49)	352 (17.4%)	48	6 (5)
2009	23,838 (2.4%)	163	12 (43)	228 (18.9%)	39	5 (4)
2010	27,907 (2.0%)	192	12 (20)	556 (5.9%)	50	5 (3)
2011	32,256 (1.4%)	148	14 (115)	283 (15.0%)	35	5 (6)
2012	36,776 (1.0%)	134	17 (124)	192 (12.4%)	37	5 (3)
Total	281,352 (1.6%)	1491		3,199 (13.7%)	477	

^a^Median number of fires per cluster and the interquartile range (IQR).

In Afghanistan there were far fewer fires detected (3702) and the annually tuned k was consistently 3. The annual number of clusters ranged from 24 to 70, with a small median number of fires per cluster across all years (between 4 to 6). The proportion of noise observations was much higher than in Iraq, ranging from 5.9% to 18.9% (Table 1). The smaller clusters and higher proportion of noise points is indicative of less tightly clustered fire signals and lower burning persistence. After excluding noise observations there were 3199 fires for analysis.

We observed a peak in the number of clustered fires in 2003, followed by a second, slightly lower peak in 2010 that aligns with the troop surge [25]. The higher number of fires in 2003 may reflect the widespread use of burn pits at newly established bases that lacked formal waste infrastructure. Early-stage burning likely involved more intense combustion, increasing the likelihood of detection. By 2010, however, the Department of Defense had begun introducing incinerators and implementing restrictions on open-air burning, potentially reducing both the frequency and intensity of detectable fires.

Notable in both Iraq and Afghanistan, in 2002 there were far fewer observations because the MODIS Aqua satellite only came online in July of that year.

### Near- and on-base burning

Of the 612 bases in Iraq, 398 (65%) had at least one fire cluster within 5 km during the study period, encompassing a total of 71,092 fires (Fig. 2, Table 2). The median number of fires in a cluster linked with a base was 20 but had a wide range from 5 to 10,235. With a median membership probability of 0.91 and small IQR (0.12), the fires near Iraqi bases were strongly associated with clusters, suggesting the core of the cluster was dense.

**Table 2:** Summary statistics (counts, medians, and interquartile ranges) of clustered fires detected within 5 km of military bases in Iraq and Afghanistan.

Country	Fires	Bases with fires	Fires per Base (range)	FRP (IQR)	Confidence (IQR)	Membership Prob (IQR)
Iraq	71,092	398 (65%)	20 (5–10,235)	14.3 (13.4)	71 (26)	0.91 (0.12)
Afghanistan	776	122 (28%)	5 (2–73)	13.2 (17.0)	61 (30)	0.16 (0.59)

**Fig. 3:**
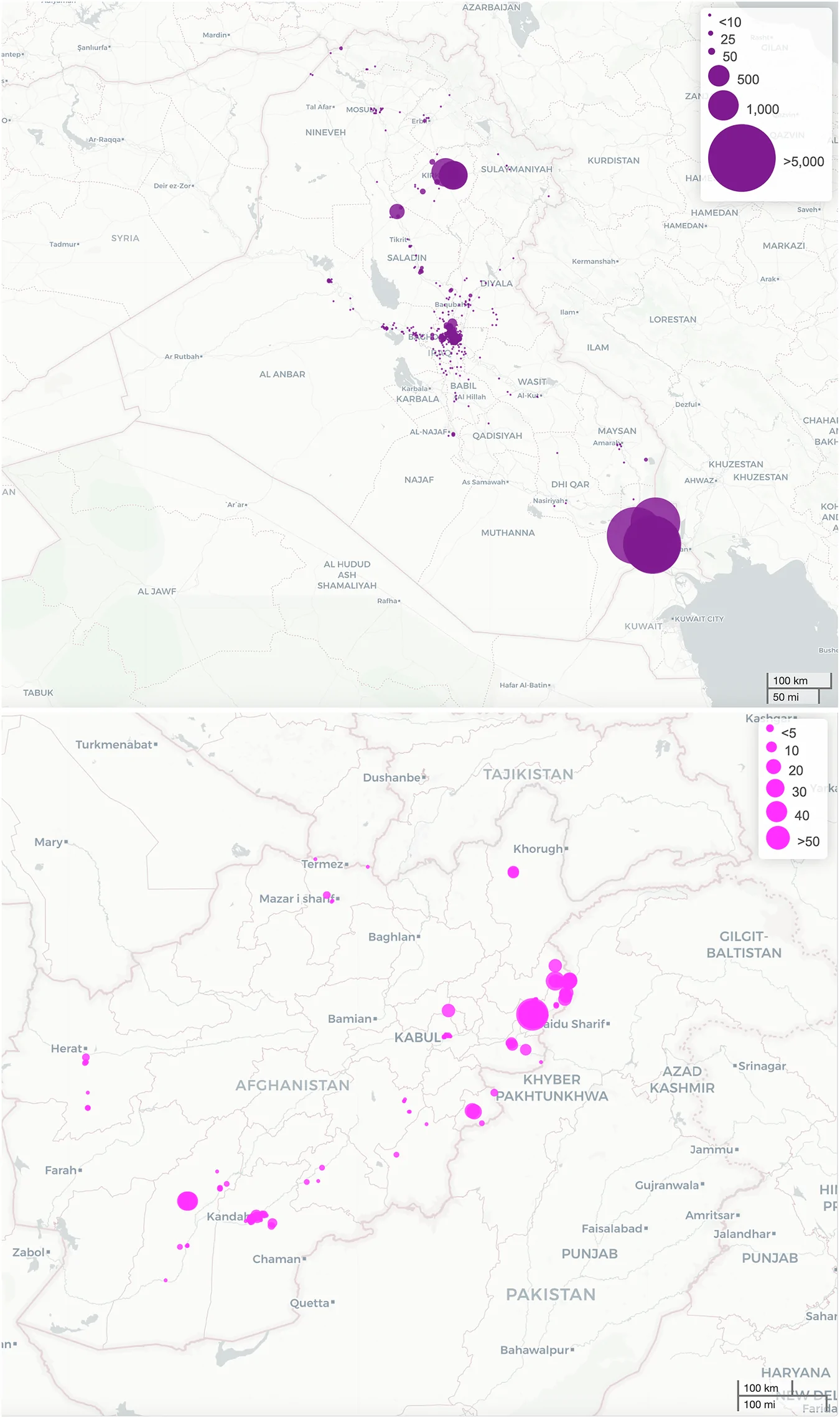
Map of the clusters of MODIS-detected fires within 5 km of bases during the study period in Iraq (top) and Afghanistan (bottom); circles are sized by the number of fires in each cluster.

Geographically, the most prominent “hotspots” in Iraq were near bases in the southern Gulf cities Basra and Az Zubayr and northern portions of the country near Baghdad, Kirkuk, and Mosul (Fig. 3).

Of the 435 bases in Afghanistan, 122 (28%) had at least one fire cluster within 5 km during the study period, encompassing a total of 776 fires (Table 2, Fig. 2). In contrast to Iraq, the median number of fires per base was smaller (5 with range 2–73) and the clusters were less tightly formed, with a median membership probability only 0.16 (IQR = 0.59). This again suggests that fire clusters in Afghanistan were more dispersed and less persistent than those in Iraq. Another notable difference was in the FRP, which was higher in Iraq (14.3 MW) than Afghanistan (13.2 MW), indicating higher temperature combustion activity in Iraq. Nevertheless, the high IQRs for each country suggest that the FRP values in both countries span a broad range, with some fires being much more intense than others, and indicative of different combustion sources.

The most prominent “hotspots” in Afghanistan were in the northeastern portions of the country on the border of Pakistan. Groupings of smaller clusters appeared in Kabul and in the south near Kandahar (Fig. 3). Consistent with how many fewer fires were detected in Afghanistan, the range of fire clusters shows far less persistence compared to Iraq.

Interestingly, despite the median retrieval confidence being similar, the median FRP of fires occurring within 5 km of military bases was lower than that of all detected fires in both Iraq and Afghanistan (Table 2). This suggests that fires near bases, which likely included burn pits, waste burning, and other localized combustion, tended to be lower in intensity compared to regional fires that included hotter combustion sources such as oil and gas flaring and refinery flaring. This difference aligns with the expectation that open-air waste burning and smoldering fires common on or near bases produce weaker thermal signatures than more industrial or landscape-scale fires.

The bases with the most persistent fire signals within 5 km were all located in Iraq, some with identified clusters across all 11 years of the study (Table 3). The most active burning locations included bases in the Gulf region of Basra, including Camp Hutch/Steelback (10,235 fires over 11 years), Manchester (9815 fires over 11 years), and Camp Al Saad (7520 fires over 11 years). To the north, Kirkuk, which is both the name of the base and the region, also showed persistent burning. Balad, the largest U.S. military base in Iraq, located outside Baghdad, had one detected cluster that persisted across 6 years of the study period.

**Table 3:** Summary statistics of the bases in Iraq and Afghanistan with largest number of clustered fires within 5 km. Number of annual clusters, number of years that satellite detections were observed, and the median fire radiative power.

Base	Iraq				Afghanistan				
	Fires	Clusters	Years	FRP	Base	Fires	Clusters	Years	FRP
Camp Hutch^a^	10,235	24	11	14.4	Phoenix^f^	75	27	7	18.0
Manchester	9815	14	11	17.9	Camp Bastion^g^	23	6	4	7.2
Camp Al Saad	7520	9	11	26.1	Warheit^h^	24	5	4	16.2
Kirkuk (K-1)	2566	22	11	17.3	Camp Clark^i^	15	6	6	12.9
Barbarian^b^	2500	22	11	12.0	Buzzard^j^	15	6	2	14.5
Al Meerah	1797	9	11	14.9	Bagram	13	6	6	10.3
Bor	648	22	11	8.6	Mustang	12	3	1	53.4
White Falcon^c^	232	11	10	7.6	Bostic	10	7	5	12.6
Baghdad-Bandit	185	7	1	7.8	Stallion	9	6	4	13.0
Iron^d^	83	4	4	12.9	Jalalabad^k^	8	4	4	7.8
Balad^e^	44	5	6	6.2					

^a^Also named Steelback, within 5 km of Chindit, Shaibah, Wessam.

^b^also named Patrol Base B within 5 km of Crazyhorse, Ashraf.

^c^also named Cop 838.

^d^also called Al Farahidy, within 5 km of Basrah, Al Jameat, Al Hussein.

^e^also named Anaconda.

^f^also called Vimoto and includes Table Rock, Atlanta/Restrepo, Korengal, Vegas, Reno.

^g^also called Camp Shorabak and Leatherneck, Tombstone, Bouresches.

^h^also called Kamdesh.

^i^within 5 km of Camp Khowst.

^j^within 5 km of Condor, Lybert, Gowardesh.

^k^also called Jalabad Airfield and includes Fob Fenty.

The bases in Afghanistan were associated with fewer fires, but many of the bases were small and within close proximity to each other. The highest concentrations of fire activity occurred in the mountainous northeast near the Pakistan border including Phoenix, which saw persistent burning over seven years, and Warheit, located 100 km to the north. Jalalabad, a city east of Kabul, also exhibited fire clusters, along with two of the largest bases in Afghanistan, Camp Bastion (west of Kandahar) and Bagram (north of Kabul), which had clusters in four to six years of the study period.

In Iraq, bases associated with oil and gas flaring such as Camp Al Saad (median FRP = 26.1 MW), had notably higher FRP than bases where on-base open-air burning was the dominant combustion source, such as Joint Base Balad (median FRP = 6.2) (Table 3). In contrast, FRP values were highly variable and inconsistent in Afghanistan, likely due to the smaller numbers of fires in clusters near the bases.

### Validation with imagery

**Fig. 4:**
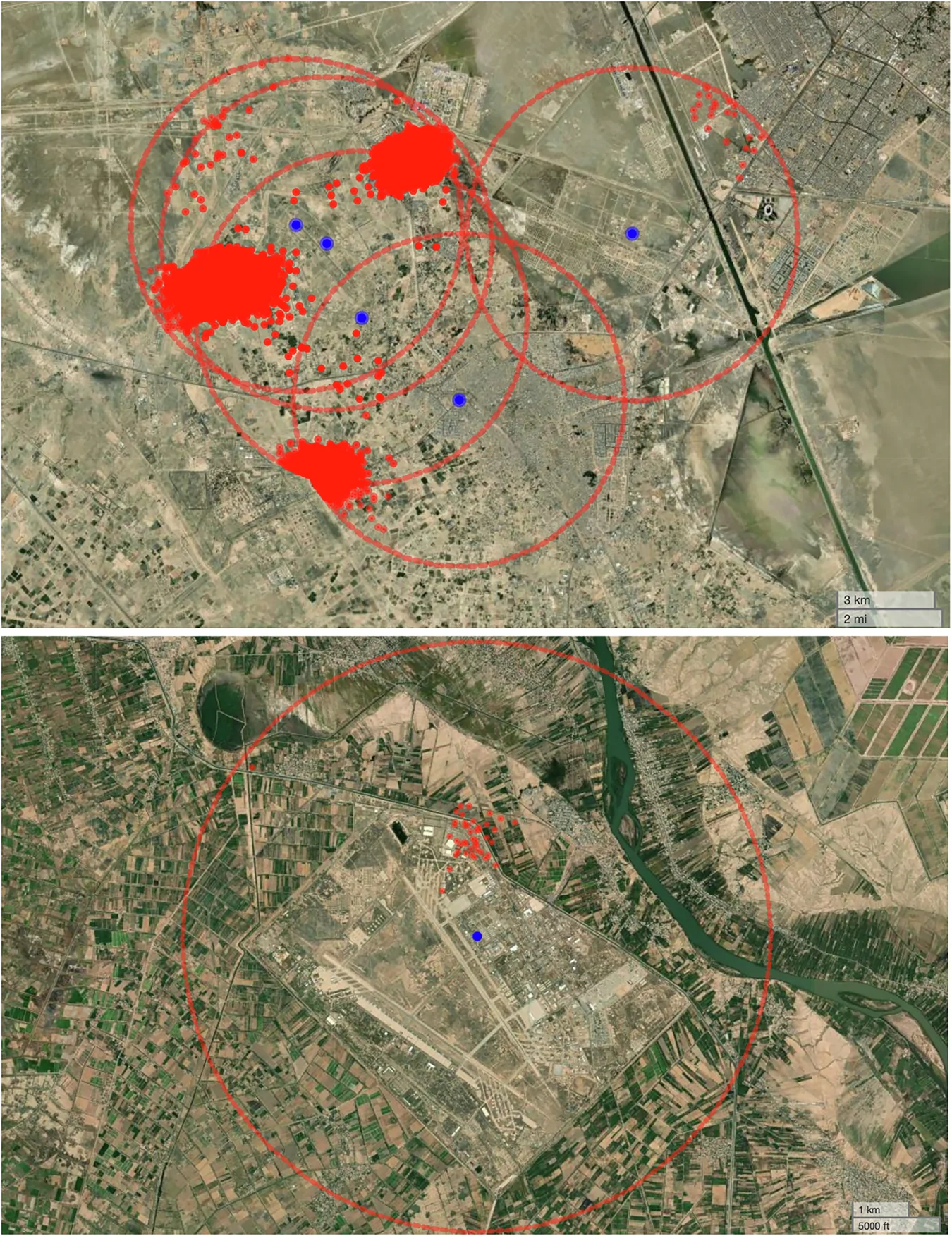
MODIS-detected fires near Camp Hutch/Steelback, Iraq (top) where nearby oil and gas dominated the fire signal, and at Joint Base Balad, Iraq (bottom) where an on-base burn pit was identified. The red dots are the MODIS-detected dires, blue dots are the georeferenced bases, and red dotted lines are the 5 km buffers around the bases.

We validated the clustered fire signals with high resolution military imagery over the study period for the bases identified in Table 2. Where available, we extracted images from Google Earth for this publication. Interesting patterns emerged between the two countries. In Iraq, we noted the most persistent burning occurred near bases in very tight clusters. For example, the 10,235 fires at Camp Hutch/Steelback, which was also near Shaibah, Chindit and Waseem, were in three clusters both on and near the base (Fig. 4). The persistent burning cluster to the south west was identified in both military imagery and GoogleEarth from 2002 to be oil and gas flaring (Fig. S1).

Similarly, with 2009 and 2010 military imagery, we confirmed a large cluster of fires just a few hundred meters to the west of Camp Al Saad. When zoomed in we identified the fire signals appearing to be from oil and gas operations and the 2010 GoogleEarth image shows active flaring (Fig. S1). K-1 (Kirkuk), close to Barbarian, had multiple sources of burning both on and off base, including oil and gas and a possible burn pit identified from 2003 military imagery (Fig. S2).

A smaller cluster of fires were observed at Joint Base Balad where we identified a burn pit in the northeast corner of the base (Fig. 4).

**Fig. 5:**
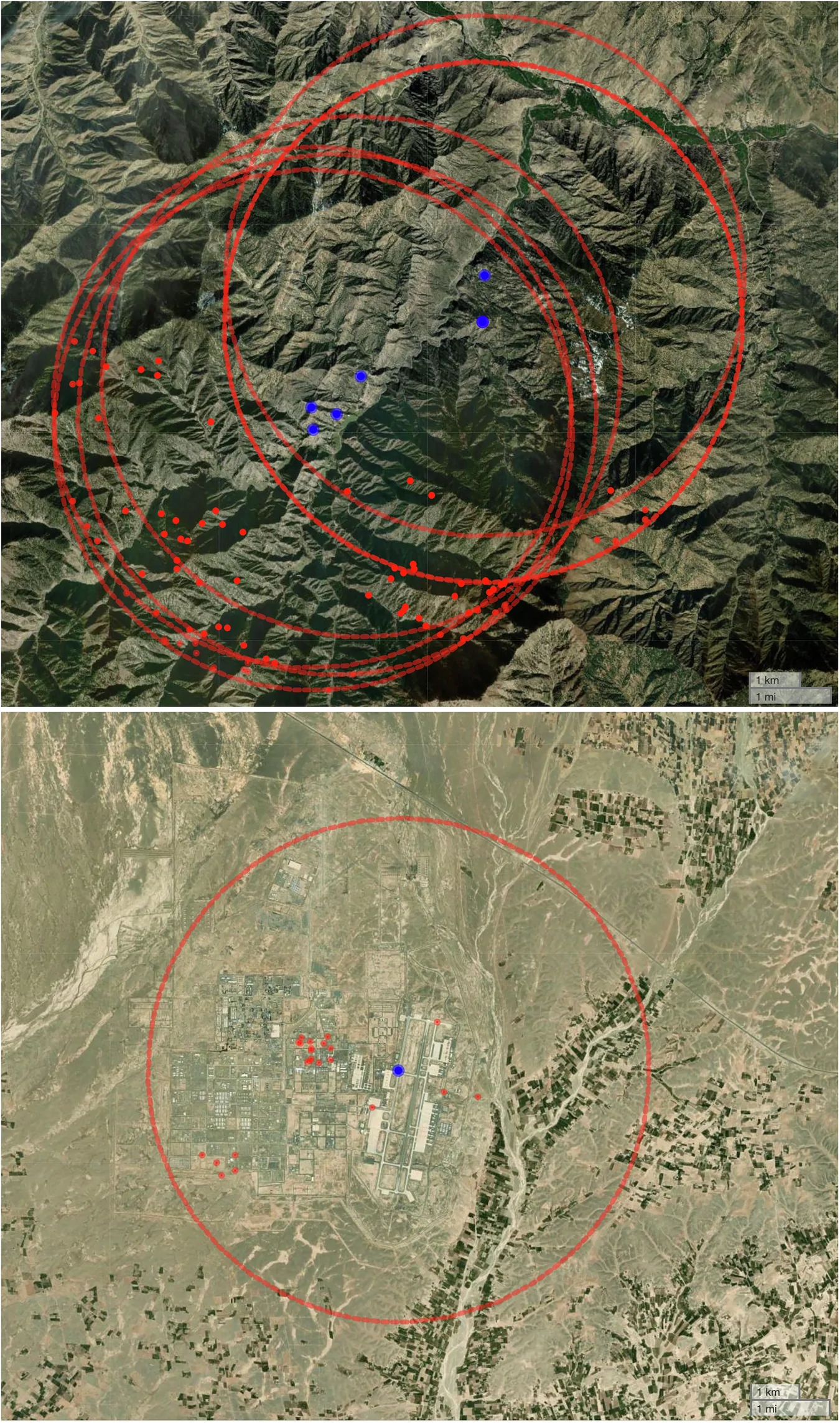
MODIS-detected fires near Phoenix (Vimoto), Table Rock, Atlanta (Restrepo), Korengal, Vegas, and Reno, Afghanistan (top), and at Camp Bastion, Afghanistan (bottom) where two on-base burn pits were identified.

As expected, fires in Afghanistan were more scattered around military bases, with fewer of the tightly formed clusters observed in Iraq. Many Afghan bases were situated in mountainous terrain, and their physical footprints were smaller. For example, although a cluster of fires was detected near Phoenix (Fig. 5), it appeared to be shared by multiple small bases near the Pakistan border. The burning in this area appeared to occur off-base and from sporadic sources. In contrast, Camp Bastion, a large airfield located further south in a sparsely populated desert region of Afghanistan showed evidence of on-base burning at two distinct locations, consistent with burn pit activity (Fig. 5). This figure also highlights inconsistencies in the military geocodes, which referred to the same base but were associated with slightly different geographic coordinates.

### Sensitivity analysis: MODIS and VIIRS

**Fig. 6:**
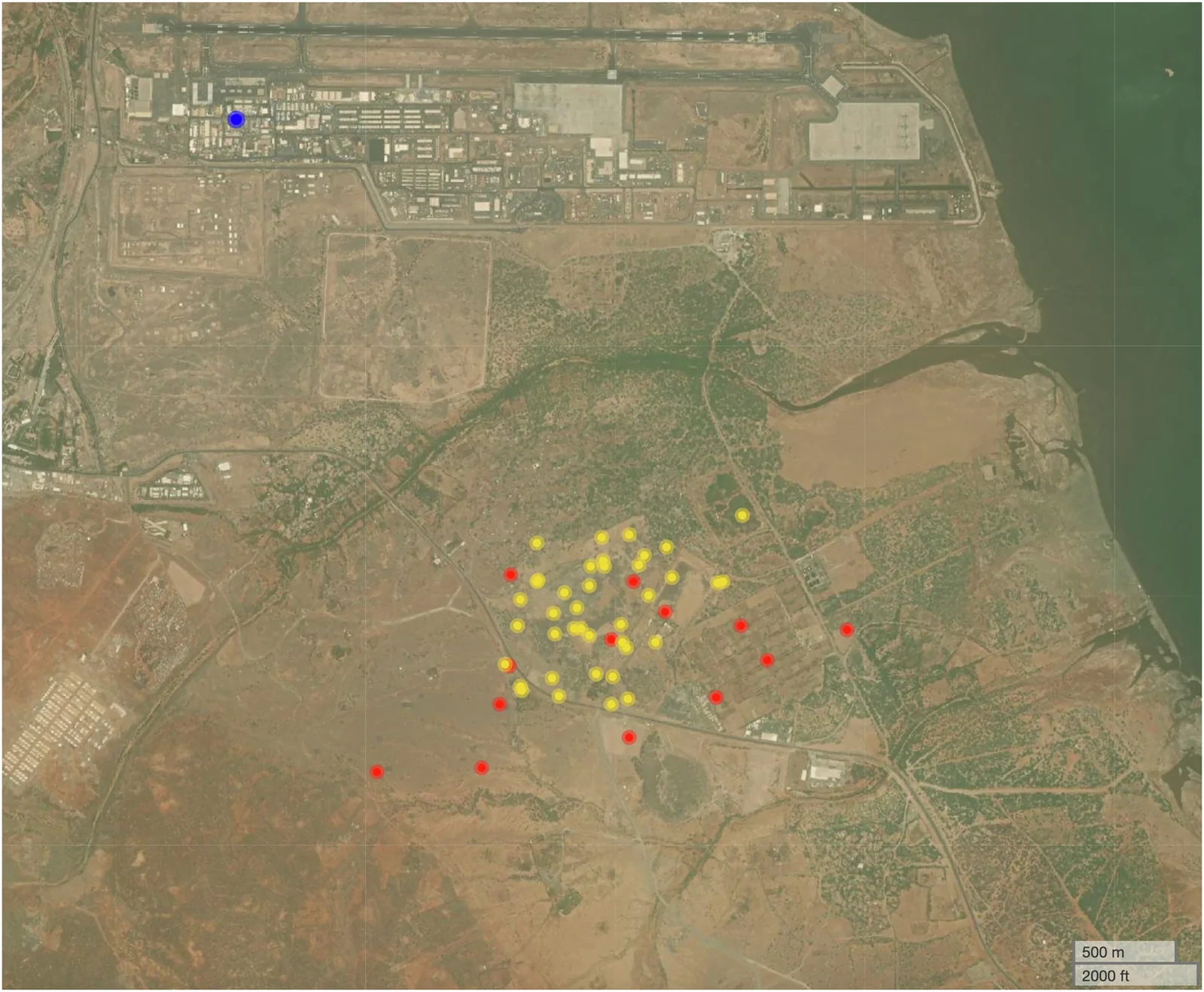
MODIS-detected fires (red dots) and VIIRS-detected fires (yellow dots) at a civilian burn pit near US military base Camp Lemonnier, Djibouti (blue dot).

The civilian burn pit, located approximately 2 km to the south of Camp Lemonnier, Djibouti was detected by both MODIS and VIIRS between 2016 and 2023 (Fig. 6). Using HDBSCAN, we identified a distinct cluster at the burn pit site. VIIRS detected 39 fires events whereas MODIS recorded only 13, a three-fold difference. This discrepancy highlights the greater sensitivity and higher spatial resolution of VIIRS compared to MODIS, particularly in detecting smaller or lower intensity fires. Notably, the median membership probability of the MODIS fires in the cluster was 0.48 compared to 0.61 for VIIRS, indicating that the VIIRS-detected fires formed a tighter and more peristent cluster. Although FRP values from MODIS and VIIRS are not directly comparable due to differences in spatial resolution, the spectral bands used by the intruments, and the retrieval algorithms, MODIS recorded a median FRP of 15.3 MW, while VIIRS had lower median FRP of 4.3 MW. This difference likely reflects the finer spatial resolution of VIIRS, which enables detection of smaller, more isolated fires with lower total radiative energy output per pixel.

## Discussion

In this study, we used satellite data to objectively identify combustion-related sources in Iraq and Afghanistan during post-9/11 military operations. While our original goal was to locate and quantify the intensity of base-related burn pits, MODIS’s limited sensitivity to lower-temperature fires made it challenging to consistently detect these smaller, smoldering sources. Nevertheless, we were able to identify likely burn pit activity at large bases (Balad, Iraq and Bastion, Afghanistan), and we confidently identified persistent off-base combustion, particularly oil and gas flaring near bases in southeast Iraq.

The heterogeneity of waste types in burn pits contributes to highly variable combustion temperatures and emission profiles [11]. This variability not only impacted our ability to detect the fires but also limited the reliability of FRP as a consistent metric, especially when comparing burn pit activity to more uniform, high-temperature sources like gas flaring.

Our findings indicate that several bases had multiple sources of combustion-related exposures, many persistent through the entire 2002–2012 deployment period. While burn pits have received significant attention through the Veterans Burn Pits Exposure Recognition Act (2022) for their toxic emissions and association with respiratory health outcomes [26], our results emphasize the importance of also accounting for other nearby pollution sources. This is important for building a more comprehensive deployment-specific exposure profile for Veterans [27].

Importantly, we are now using the location, timing, and persistence of fire detections to construct deployment-specific exposure metrics that offer a level of detail not previously available to researchers or clinicians examining Veteran health [9]. Historically, exposure assessment in this population has relied on self-reported questionnaire data, including general recollections of exposure to burn pit smoke, vehicle exhaust and other open-air combustion sources [2]. In contrast, the quantitative satellite-based exposure metrics developed here, such as fire cluster frequency, duration, spatial extent, and proximity to bases, will provide objective and reproducible measures of potential exposure. Evidence from environmental epidemiology supports this approach. For example [28], demonstrated that VIIRS-detected flaring was a better predictor of health outcomes than simple distance-based metrics in an oil and gas region of Texas.

Deployment histories collected as part of CSP#595 include detailed records of base names, service periods, and time frames for each Veteran in the study, enabling the development of base-level, time-resolved exposure metrics. These metrics may include presence of on- and near-base fire clusters; distance from the base to each identified fire cluster within 5 km (which can be weighted by the inverse of distance and summed to account for exposure to multiple clusters); the number of fires detected on or near a base during deployment; and the median fire intensity (e.g., FRP) associated with those detections.

In previous work, we showed that visibility measurements coupled with satellite aerosol optical depth could be used to reliably estimate PM_2.5_ in deployment areas without ground monitoring [29]. The ability to map combustion sources from satellite data now provides a critical missing piece—a method to attribute PM_2.5_ to specific fire sources. Similar approaches have been successfully applied to wildfire-specific PM_2.5_ exposure modeling in the U.S. [30].

While satellite detection of biomass burning has become routine [19], uncertainties remain. As we observed, MODIS struggles to discern small and low-temperature or smoldering fires such as burn pits due to their weaker thermal signatures [31]. Moreover, FRP, although useful for estimating combustion energy, has significant uncertainty in characterizing fire types and intensities [32]. This limits the capabilities of FRP to distinguish between flaming and smoldering combustion, a key distinction when evaluating exposure toxicity [33]. In addition, the limited number of detected fire clusters in certain years, particularly in Afghanistan, may constrain the ability to generate consistent annual exposure metrics across all bases, reducing temporal resolution and statistical power in epidemiologic analyses.

Despite these limitations, our analyses revealed that median FRP of MODIS fires at Joint Base Balad, where there was a burn pit, was over 13.2 MW lower than the FRP at Camp Al Saad where we confirmed the presence of oil and gas flaring with imagery. We observed an under-detection of smaller fires due to the 1 km resolution of MODIS and poorer specificity at lower temperature detections. In contrast, VIIRS, with 375 m resolution and enhanced spectral bands for more robust detections, detected more than three times as many fire events at a civilian burn pit near Camp Lemonnier, Djibouti. These findings are consistent with [34] who reported that VIIRS active fire detected five to ten times more fire pixels MODIS during simultaneous overpasses.

Future work could explore integrating VIIRS-based fire detection patterns to improve interpretation of MODIS data retrospectively. Calibration models or correction factors that help infer likely fire activity patterns during the earlier MODIS-only period could be developed using machine learning or statistical modeling approaches to quantify relationships between MODIS and VIIRS detections under similar geographic and environmental conditions. These models could then be applied to MODIS fires to estimate the number, location, and type of fires that were likely missed due to resolution and sensitivity limitations.

To our knowledge, this is the first study to use satellite-detected fires to identify and characterize combustion sources near US military bases during post-9/11 operations. We found persistent burning from multiple sources including burn pits, oil and gas flaring, and other open-air combustion on and near military bases in Iraq and Afghanistan from 2002 to 2012. The resulting exposure metrics provide a novel, objective framework for assessing Veterans’ combustion-related exposures and will support ongoing research into the long-term health impacts of deployment.

## Supplementary information

## Data Availability

The MODIS and VIIRS satellite fire detection data, along with the code used for HDBSCAN clustering in this study, are available on the GitHub repository: https://github.com/meredithfranklin/MODIS-VIIRS-fires. Unlinked MODIS and VIIRS satellite data for Iraq and Afghanistan are from public data sources but are available from the corresponding author upon request. Geospatial information for military bases is protected and requires U.S. Department of Veterans Affairs approval for release.
