# Supplementary material for: Exposures to combustion sources near military operations in Iraq and Afghanistan using satellite observations
Source: J Expo Sci Environ Epidemiol. 2025 Oct 10;36(5):834–44. doi: 10.1038/s41370-025-00804-z (PMC12637216; doi:10.1038/s41370-025-00804-z)
Supplement: Supplementary file 1 [file 41370_2025_804_MOESM1_ESM.docx]

Supplementary Information

**Figure S1.** Top: MODIS-detected fires (red dots) at Camp Al Saad, Iraq with Google Earth image from 2010; Bottom: MODIS-detected fires (red dots) at Camp Hutch, Iraq, with Google Earth image from 2002.

**Figure S2.** MODIS-detected fires (red dots) at Kirkuk and Barbarian bases (blue dots) showing clusters of off-base burning from oil and gas operations and scattered on-base burning from a burn pit (Google Earth image from 2004).
