# Supplementary material for: Exposures to combustion sources near military operations in Iraq and Afghanistan using satellite observations
Source: J Expo Sci Environ Epidemiol. Author manuscript; Available in PMC 2025 Nov 21. (PMC12637216; doi:10.1038/s41370-025-00804-z)
Supplement: Supplementaer [file NIHMS2117652-supplement-Supplementaer.docx]

Supplementary Information


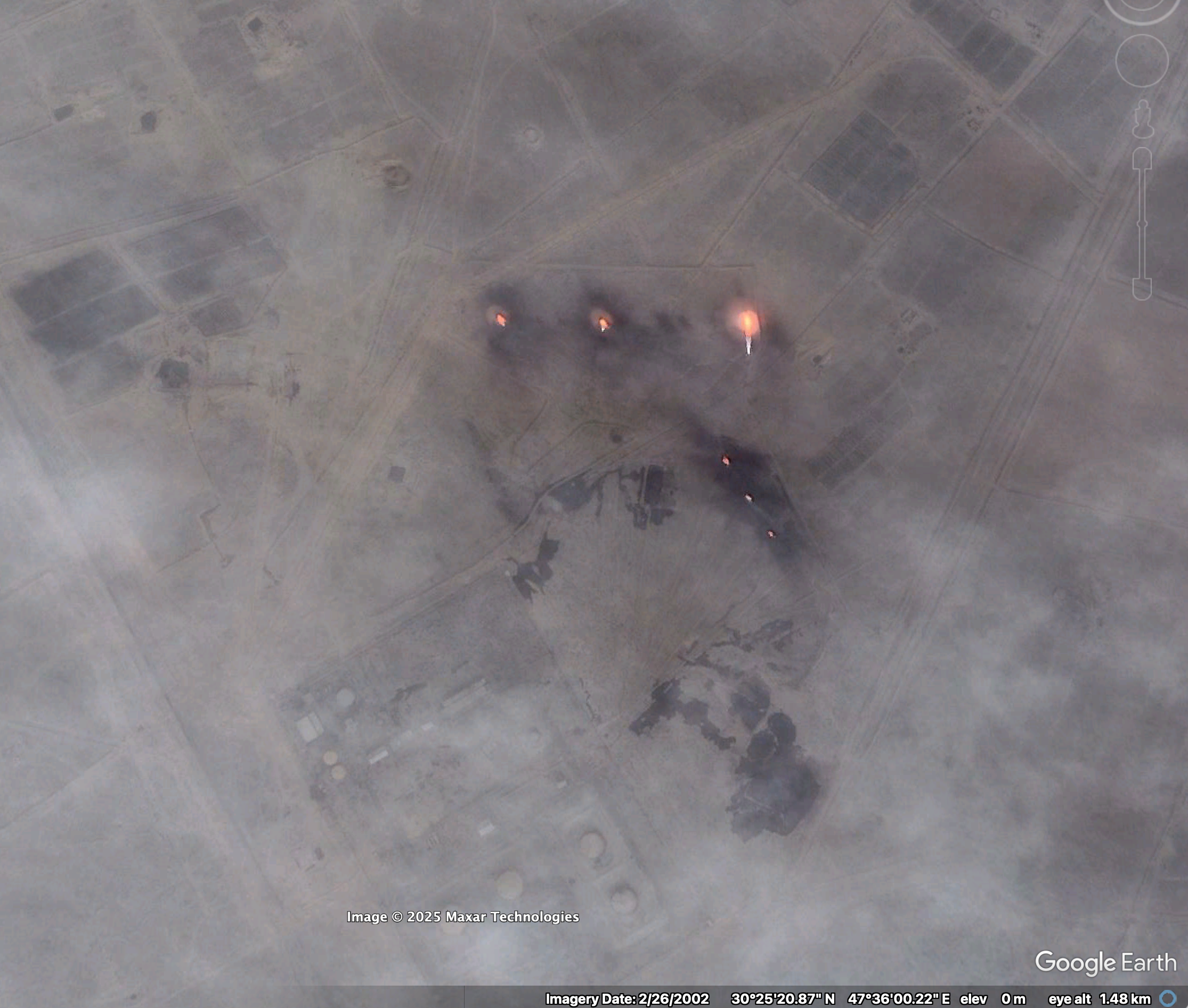


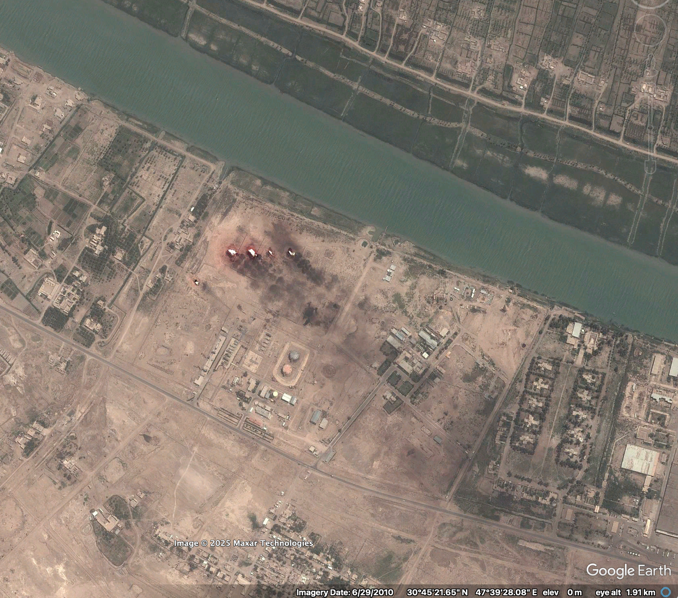


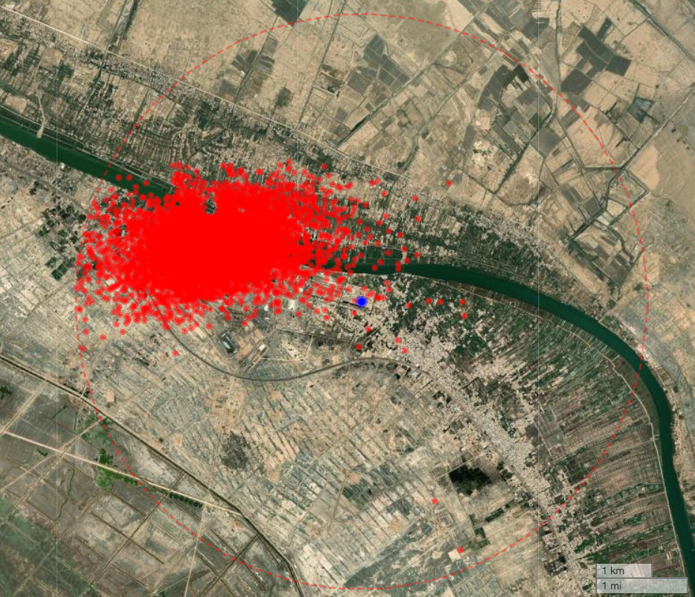


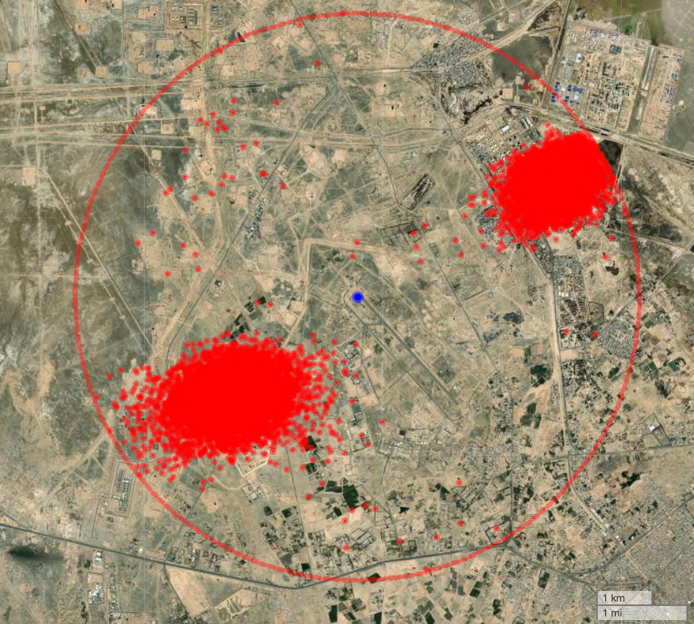


**Figure S1.** Top: MODIS-detected fires (red dots) at Camp Al Saad, Iraq with Google Earth image from 2010; Bottom: MODIS-detected fires (red dots) at Camp Hutch, Iraq, with Google Earth image from 2002.


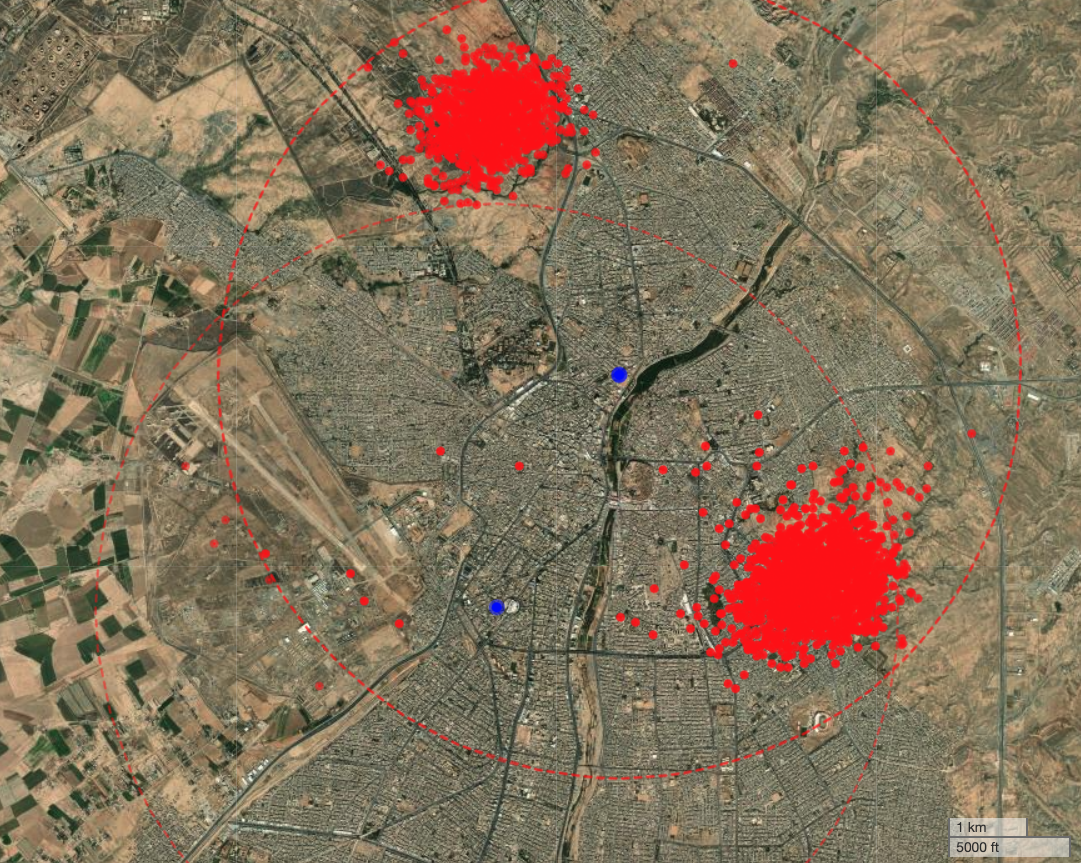


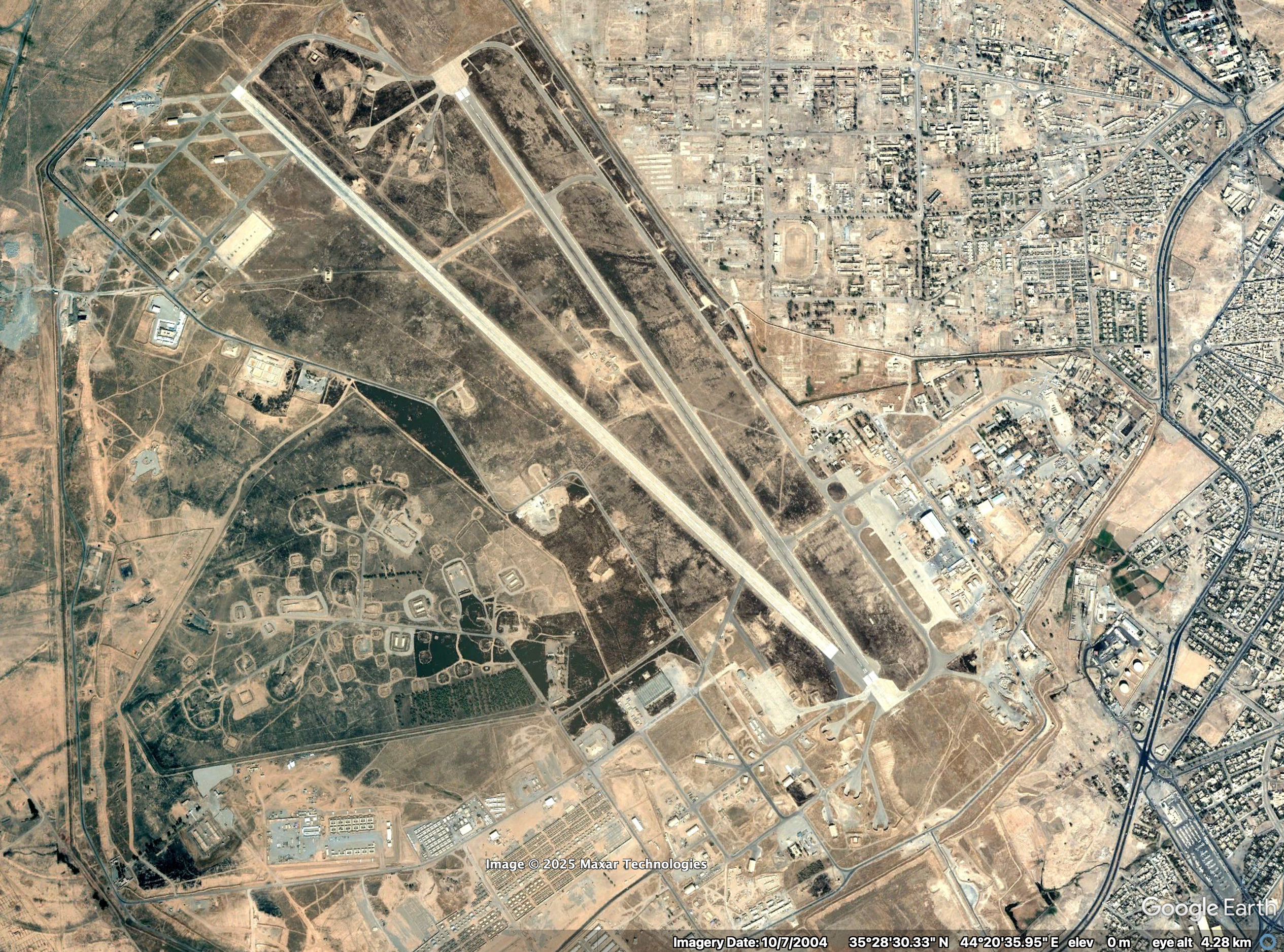


**Figure S2.** MODIS-detected fires (red dots) at Kirkuk and Barbarian bases (blue dots) showing clusters of off-base burning from oil and gas operations and scattered on-base burning from a burn pit (Google Earth image from 2004).
